# Data for the crystal structure of APRIL–BAFF–BAFF heterotrimer

**DOI:** 10.1016/j.dib.2015.12.024

**Published:** 2015-12-18

**Authors:** Klaus Maskos, Alfred Lammens, Seng-Lai Tan, Henry Hess, Wolf Palinsky, Pascal Schneider, Xuliang Jiang

**Affiliations:** a Proteros Biostructures GmbH, D-82152 Planegg, Germany; b EMD Serono Research & Development Institute, Billerica, MA 01821, U.S.A; c Merck KGaA, D-64293 Darmstadt, Germany; d Merck Serono S.A., Aubonne CH-1170, Switzerland; e Department of Biochemistry, University of Lausanne, CH-1066 Epalinges, Switzerland

## Abstract

The TNF family ligands B cell activation factor (BAFF) and a proliferation-inducing ligand (APRIL) modulate B cell function by forming homotrimers and heterotrimers. To determine the structure of a heterotrimer of BAFF and APRIL, these ligands were expressed as a single chain protein in HEK 293 cells, purified by affinity and size exclusion chromatographies, and crystallized. Crystals belonging to the orthorhombic crystal system with a space group of C222_1_ diffracted to 2.43 Å. Initial structural solution was obtained by the molecular replacement method, and the structure was further refined to an *R* factor of 0.179 and free *R* factor of 0.234. The atomic coordinates and structure factors have been deposited into the Protein Data Bank (accession code 4ZCH).

**Specifications table**

**Table:** 

Subject area	*Biology*
More specific subject area	*Structural biology*
Type of data	*Table, image*
How data was acquired	*Data was collected at Swiss Light Source (SLS) Beamline PXI/X06SA. Detector type: Pilatus 6 M manufactured by DECTRIS. Data integration was done using the software XDS and XSCALE*
Data format	*.cbf image files, .mtz processed file, .lp integration and scale files*
Experimental factors	*The APRIL–BAFF–BAFF heteotrimer was created by linking APRIL and BAFF subunits by five amino acid residues linkers (GGGGS)*
Experimental features	*The single chain APRIL–BAFF–BAFF heterotrimer was secreted from HEK 293 cells and purified by affinity chromatography and size exclusion chromatography. The purified protein was crystallized and the crystal diffracted to 2.43 Å resolution. The structure was solved by the molecular replacement method*
Data source location	*Proteros Biostructures GmbH, D-82152 Planegg, Germany and EMD Serono Research & Development Institute, Billerica, MA, USA*
Data accessibility	*Deposited to the RCSB Protein Data Bank* (http://www.rcsb.org). *Accession code RCSB PDB: 4ZCH*

**Value of the data**

• The SDS-gel data is a contribution to the pool of similar data from others to show, semi-quantitatively, the purity of protein samples for crystallization.
• The crystallization condition can be collected by others in designing better matrix solutions for protein crystallization.
• Our procedures and method, including the input parameters and output statistics of the reflection measurements, can be compared with those used by others in the field for establishing a best practice.

## 1 Data

BAFF and APRIL belong to a family of closely related TNF family ligands [1], [2]. Although crystal structures of BAFF or APRIL homotrimers are known since several years, we only recently reported the crystal structure of BAFF and APRIL heterotrimers [3]. In order to generate a homogeneous protein material for structural studies, we joined one APRIL and two BAFF subunits into a single chain protein, by introducing two glycine-serine linkers (GGGGS) in between ligand subunits. The expressed protein APRIL–BAFF–BAFF was crystalized and its X-ray diffraction structure was solved and deposited into Protein Data Bank with accession code 4ZCH [3].

## 2 Experimental design, materials and methods

### 2.1 Protein production

The single-chain heterotrimer was constructed by linking one APRIL to two BAFF molecules. It started from an N-terminal Ig secretion signal (MNFGFSLIFLVLVLKG), a His_6_ (HHHHHH)-FLAG (DDYKDDDDK) tag, followed by a TEV cleavage site (ENLYFQ), a human APRIL subunit (amino acid residues 111-250) with a T126A mutation, a GGGGS linker, a human BAFF subunit (amino acid residues 140-285), then another GGGGS linker, and a C-terminal second human BAFF subunit (amino acid residues 140-285). Mutation T126A was introduced to remove a potential glycosylation site of APRIL. This mature single chain heteromer has the formula of [His_6_-FLAG-TEV-GS-hAPRIL(aa111-250, T126A)-GGGGS-hBAFF(aa140-285)-GGGGS-hBAFF(aa140-285)]. The constructed single chain was expressed in HEK293 cells with a yield of 250 μg/L.

**Fig. 1:**
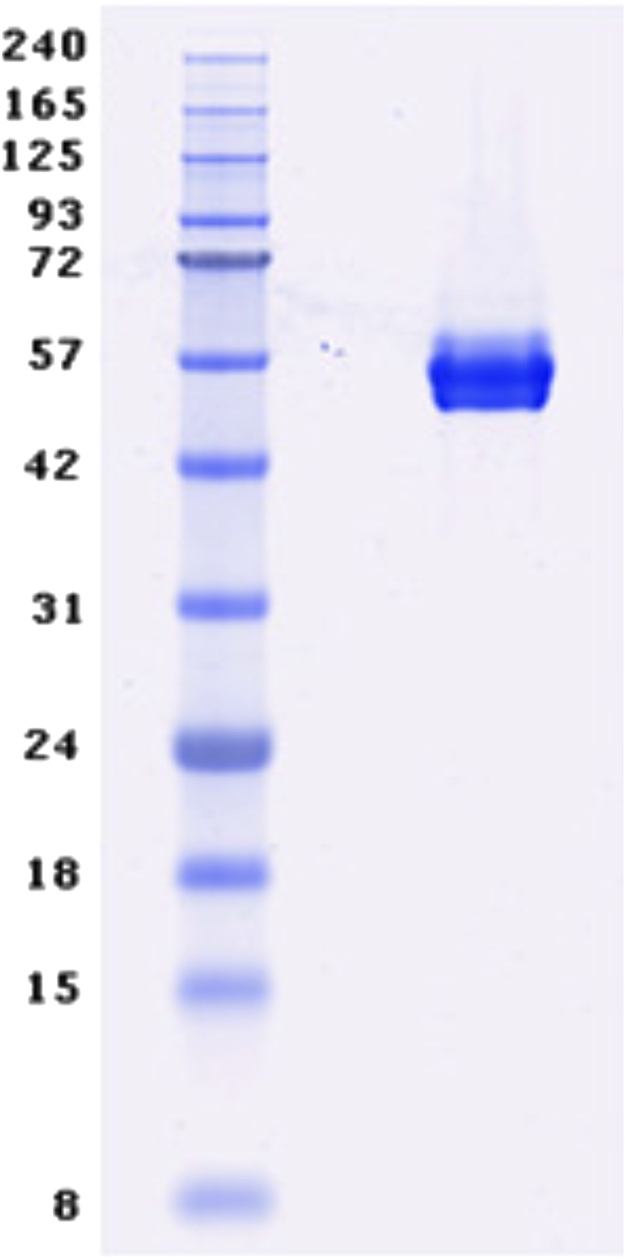
SDS-PAGE analysis of the purified APRIL–BAFF–BAFF protein sample. The molecular-weight protein ladder is on the left, the protein sample on the right.

The purification was carried out first by affinity chromatography on nickel-nitrilotriacetic acid, then tag was cleaved with tobacco etch virus protease, and the protein was further purified by size exclusion chromatography on a Superdex-200 column. The purified protein solution in 20 mM HEPES/NaOH pH 7.5 and 150 mM NaCl was concentrated using a 30 kD ultrafiltration device (Vivascience) to a concentration of 14 mg/mL, as determined by Nanodrop UV–vis spectrophotometry. Fig. 1 shows the purified protein sample analyzed by SDS-PAGE and Coomassie blue staining. The apparent molecular weight of the protein was around 50 kDa.

## 3 Crystallization

The purified protein was crystallized by trying various pH conditions and other crystallization factors. The hit conditions were optimized to obtain crystals suitable for X-ray diffraction measurements. Crystals were obtained by the hanging drop vapor diffusion method incubated at 20 °C. Protein solution at 6 mg/ml in 20 mM Hepes/NaOH pH 7.5, 150 mM NaCl (0.5 µl) was mixed with 0.5 μl of a reservoir solution of 0.1 M Tris/HCl, pH 8.75, 14% PEG6000 (w/v), 1 M LiCl in a 1-to-1 ratio. Once obtained, crystals were mixed with reservoir solution supplemented with 10% (v/v) 2,3-butanediol prior to flash freezing in liquid nitrogen.

## 4 Data collection and processing

The diffraction data were collected at 100 °K at X-ray wavelength of 0.99998 Å at beamline X06SA/Swiss Light Source (SLS) using a Pilatus 6 M detector, and integrated using the software XDS and XSCALE [4]. Table 1 shows the parameters used in the data collection. The crystal system was determined to be orthorhombic with space group C222_1_. Its unit cell dimensions were of 57.04 Å, 117.86 Å and 295.52 Å.

Data were processed to 2.43 Å resolution. A total of 134,837 reflections were measured, referring to 36,901 unique reflections, representing a completeness of 96.7% and a redundancy of 3.7. The average signal to noise ratio was 13.46 for the whole data set and 3.06 for the highest resolution shell (2.68–2.43 Å). The data set quality is further assessed by two quantities, *R*_sym_ and *R*_merge_, in order to measure internal agreement (residual factors) of symmetry-related reflections and redundant data. The *R*_sym_ and *R*_merge_ were 7.9% and 9.2%, respectively. Table 2 shows the correlation between observed and expected profiles, and Table 3, Table 4 show the *R*-factors and Wilson statistics of the data set, respectively.

## 5 Structure modeling and refinement

The structural phase information was initially obtained by the molecular replacement method, by using the software Phaser in CCP4 [5], [6]. The published structures of APRIL (PDB accession code: 1Q5X) and BAFF (PDB code: 1KD7) were used as search models. About 3% of the measured reflections were excluded for the calculation of the free *R*-factor in order to cross-validate the correctness of the final model. Subsequent model building was done in multiple rounds using software COOT. Refinement was performed using the REFMAC5 software with bulk solvent correction and TLS parameterization in the CCP4 package [6], [7], [8], [9]. The water model was built with the “Find waters” algorithm of COOT by putting water molecules in peaks of the Fo–Fc map contoured at 3.0 sigma, followed by refinement with REFMAC5 and checking all waters with the validation tool of COOT. The occupancy of side chains, which were in negative peaks in the Fo–Fc map (contoured at −3.0 sigma), were set to zero. The model was further subjected to the refinement using software BUSTER [10]. The final refinement residual factors, *R*_work_ and *R*_free_, are 17.9% and 23.4%, respectively. The r.m.s. deviations for bond length and bond angle are 0.01 Å and 1.22°, respectively. The Ramachandran Plot of the final model shows 95% of all residues in the favored region, and 0.5% in the outliers region [11] and is in agreement with the main-chain conformational tendencies shown in an earlier study [12].

## 6 The deposited data

The structure contains two APRIL–BAFF–BAFF heterotrimers in each asymmetry unit. A total of 6997 atoms (6650 from protein, 339 from water and 8 from a TRIS buffer molecule) were included in the final model. Table 5 is the list of amino acid residues in the final model and their corresponding amino acids in the natural mature protein [13]. The atomic coordinates and structure factors have been deposited into the Protein Data Bank (http://www.rcsb.org) with the accession code 4ZCH.

## Figures and Tables

**Table 1:** Input parameters in the data collection.

**Input parameter**	**Value**
Number of space groups used in Integrate step	1
Unit cell constants used by Integrate	57.182, 65.595, 295.885, 89.990, 90.001, 64.271
Friedel׳s_Law	TRUE
Profile_Fitting	TRUE
Overload	1,048,500
MINPK	75.00000
WFAC1	1.0
Include_Resolution_Range	50.000, 2.430
Data_Range	1 201
Rotation_Axis	0.999998 −0.000013 −0.001892
Oscillation_Range	0.50000
Starting_Angle= 0.000	0.000
X-ray_Wavelength	0.99998
Incident_Beam_Direction	−0.003068 0.002974 1.000011
Fraction_of_Polarization	0.99
Polarization_Plane_Normal	0.000000 1.000000 0.000000
Air	0.00034
Detector	PILATUS
Silicon, Sensor_Thickness	3.942633, 0.320000
Number of Detector Segments	1
NX, NY, QX, QY	2463, 2527, 0.172000, 0.172000
ORGX, ORGY	1166.43, 1256.77
Detector_Distance	390.173
Direction_of_Detector_*X*-axis	1.00000 0.00000 0.00000
Direction_of_Detector_*Y*-axis	0.00000 1.00000 0.00000
Beam_Divergence_E.S.D.	0.044
Reflecting_Range_E.S.D.	0.227
Minimum_ZETA	0.050
Maximum_Error_of_Spot_Position	3.0
Maximum_Error_of_Spindle_Position	2.0
Minimum_I/Sigma	3.0
Reflections/Correction_Factor	50
Strict_Absorption_Correction	False
Absorption Corrections	Decay modulation

**Table 2:** Correlation between observed and expected profiles.

**I/Sigma**^a^	**CORR**^b^	**E.S.D.**^c^	**<I>**^d^	**Number**^e^
−3...−2	−0.187	0.067	−191	566
−2...−1	−0.136	0.036	−82	3252
−1... 0	−0.087	0.034	−20	50,640
0...1	0.102	0.040	28	117,952
1...2	0.181	0.055	81	60,013
2...3	0.282	0.075	137	29,554
3...6	0.409	0.116	241	48,658
6...9	0.560	0.126	403	25,037
9...12	0.639	0.120	551	15,496
12...15	0.673	0.118	707	11,457
15...18	0.688	0.118	869	8698
18...21	0.689	0.118	1048	6602
21...24	0.681	0.124	1231	5217
24...27	0.668	0.126	1443	4148
27...30	0.648	0.134	1663	3130
30...33	0.623	0.134	1853	2440
33...36	0.599	0.138	2052	1801
36...39	0.561	0.138	2307	1391
39...42	0.520	0.137	2613	967
42...45	0.466	0.132	3300	690
45...48	0.416	0.121	5376	394
48...51	0.337	0.088	10,089	91

a I/Sigma=mean of intensity/*σ*, where *σ*^2^=4.0*[variance(I; from counting statistics)+0.0001*I^2^]

b CORR=mean correlation factor between observed and expected reflection profiles.

c E.S.D.=estimated standard deviation of CORR.

d <I>=mean LP-corrected reflection intensity, assuming unpolarized incident beam.

e Number=number of accepted reflections used to calculate I/Sigma, CORR, E.S.D., and <I>.

**Table 3:** *R*-factor statistics for intensities of the processed data set.

**Resolution**	***R*****-factor**	***R*****-factor**	**Compared**
**limit**	**observed (%)**	**expected (%)**	
15.73	1.9	2.2	506
9.83	1.9	2.3	1400
6.74	2.6	3.0	4305
5.24	3.8	4.1	6924
4.14	3.4	3.8	13,574
3.39	5.9	6.1	21,402
2.96	12.5	13.1	25,893
2.68	26.0	26.8	24,314
2.43	50.5	52.2	34,281
Total	7.9	8.3	132,599

**Table 4:** Wilson statistics of scaled data set.^a^

**#**	**RES**	**SS**	**<I>**	**log (<I>)**	**BO**

502	13.510	0.001	1.5217E+06	14.235	122.3
850	8.455	0.003	1.0609E+06	13.875	99.5
1115	6.621	0.006	5.8402E+05	13.278	113.3
1266	5.627	0.008	6.2625E+05	13.348	77.4
1407	4.967	0.010	1.1441E+06	13.950	30.6
1607	4.503	0.012	1.2774E+06	14.060	20.7
1735	4.142	0.015	1.0029E+06	13.818	25.8
1858	3.858	0.017	7.1448E+05	13.479	32.5
1857	3.625	0.019	6.1713E+05	13.333	32.5
2002	3.431	0.021	4.2794E+05	12.967	37.8
2177	3.264	0.023	3.1404E+05	12.657	40.8
2291	3.120	0.026	2.2558E+05	12.326	43.7
2380	2.993	0.028	1.6856E+05	12.035	45.4
2458	2.881	0.030	1.2718E+05	11.753	46.8
2427	2.781	0.032	1.0851E+05	11.595	46.0
2632	2.690	0.035	8.3990E+04	11.338	46.8
2690	2.606	0.037	6.2624E+04	11.045	47.9
2795	2.532	0.039	4.8680E+04	10.793	48.4
2852	2.462	0.041	4.3740E+04	10.686	47.1

a Data is divided into resolution shells and a straight line, A-2*B*SS is fitted to log <I>, where, RES=mean resolution (Angstrom) in shell. SS=mean of (sin(THETA)/LAMBDA)^2^ in shell. <I>=mean reflection intensity in shell. BO=(A – log<I>)/(2*SS). #=number of reflections in resolution shell. Wilson line (using all data): A=14.570 B=44.922 CORRELATION=0.95.

**Table 5:** List of amino acid positions in the natural mature protein or expression construct (Uniprot database numbers Q9Y275 for BAFF and O75888 for APRIL) and their corresponding positions in the final structure.

	**Mature protein chain (Uniprot)**	**In expression construct (Uniprot number)**	**Amino Acids in structure**		
**chain**	**(Uniprot number)**	**(PDB (4ZCH) number)**
human APRIL	105-250	111-250	A/B	115-250	7-142
human BAFF	134-285	140-285	A	142-285	150-293
301-444
B	143-285	151-293
141-285	300-444
Mutation	T126 (APRIL)	A126 (APRIL)	A/B	A126	A18
G4S linker	−	−	A/B	−	294
