# Supplementary material for: Data for the crystal structure of APRIL–BAFF–BAFF heterotrimer
Source: Data Brief. 2015 Dec 18;6:438–44. doi: 10.1016/j.dib.2015.12.024 (PMC4712316; doi:10.1016/j.dib.2015.12.024)
Supplement: Supplementary file 1 — Supplementary material [file mmc1.pdf]

AUTHOR DECLARATION FORM

We wish to draw the attention of the Editor to the following facts which may be considered as potential conflicts of interest and to significant financial contributions to this work.

K. M. and A. L. are employees of Proteros Biostructures GmbH. S.-L. T., H. H., W. P. and X. J. are employees of Merck, KGaA or its subsidiary, EMD Serono. P. S. is supported by a research grant from EMD Serono, a subsidiary of Merck, KGaA.

We confirm that the manuscript has been read and approved by all named authors and that there are no other persons who satisfied the criteria for authorship but are not listed. We further confirm that the order of authors listed in the manuscript has been approved by all of us.

We confirm that we have given due consideration to the protection of intellectual property associated with this work and that there are no impediments to publication, including the timing of publication, with respect to intellectual property. In so doing we confirm that we have followed the regulations of our institutions concerning intellectual property.

We understand that the Corresponding Author is the sole contact for the Editorial process (including Editorial Manager and direct communications with the office). He/she is responsible for communicating with the other authors about progress, submissions of revisions and final approval of proofs. We confirm that we have provided a current, correct email address which is accessible by the Corresponding Author and which has been configured to accept email from xuliang.jiang@emdserono.com.

Signed by all authors as follows:

**Name**	**Signature**	**Date**
Klaus Maskos		
Alfred Lammens		
Seng-Lai Tan		
Henry Hess		
Wolf Palinsky		
Pascal Schneider	P. Schneider	Sept 19th, 2015
Xuliang Jiang		

AUTHOR DECLARATION FORM

We wish to draw the attention of the Editor to the following facts which may be considered as potential conflicts of interest and to significant financial contributions to this work.

> K. M. and A. L. are employees of Proteros Biostructures GmbH. S.-L. T., H. H., W. P. and X. J. are employees of Merck, KGaA or its subsidiary, EMD Serono. P. S. is supported by a research grant from EMD Serono, a subsidiary of Merck, KGaA.

We confirm that the manuscript has been read and approved by all named authors and that there are no other persons who satisfied the criteria for authorship but are not listed. We further confirm that the order of authors listed in the manuscript has been approved by all of us.

We confirm that we have given due consideration to the protection of intellectual property associated with this work and that there are no impediments to publication, including the timing of publication, with respect to intellectual property. In so doing we confirm that we have followed the regulations of our institutions concerning intellectual property.

We understand that the Corresponding Author is the sole contact for the Editorial process (including Editorial Manager and direct communications with the office). He/she is responsible for communicating with the other authors about progress, submissions of revisions and final approval of proofs. We confirm that we have provided a current, correct email address which is accessible by the Corresponding Author and which has been configured to accept email from xuliang.jiang@emdserono.com.

Signed by all authors as follows:

**Name**	**Signature**	**Date**
Klaus Maskos		
Alfred Lammens		
Seng-Lai Tan		
Henry Hess		
Wolf Palinsky		
Pascal Schneider		
Xuliang Jiang	[illegible]	9/22/2015

AUTHOR DECLARATION FORM

We wish to draw the attention of the Editor to the following facts which may be considered as potential conflicts of interest and to significant financial contributions to this work.

K. M. and A. L. are employees of Proteros Biostructures GmbH. S.-L. T., H. H., W. P. and X. J. are employees of Merck, KGaA or its subsidiary, EMD Serono. P. S. is supported by a research grant from EMD Serono, a subsidiary of Merck, KGaA.

We confirm that the manuscript has been read and approved by all named authors and that there are no other persons who satisfied the criteria for authorship but are not listed. We further confirm that the order of authors listed in the manuscript has been approved by all of us.

We confirm that we have given due consideration to the protection of intellectual property associated with this work and that there are no impediments to publication, including the timing of publication, with respect to intellectual property. In so doing we confirm that we have followed the regulations of our institutions concerning intellectual property.

We understand that the Corresponding Author is the sole contact for the Editorial process (including Editorial Manager and direct communications with the office). He/she is responsible for communicating with the other authors about progress, submissions of revisions and final approval of proofs. We confirm that we have provided a current, correct email address which is accessible by the Corresponding Author and which has been configured to accept email from xuliang.jiang@emdserono.com.

Signed by all authors as follows:

Name	Signature	Date
Klaus Maskos		
Alfred Lammens		
Seng-Lai Tan		09/18/15
Henry Hess		
Wolf Palinsky		
Pascal Schneider		
Xuliang Jiang		

AUTHOR DECLARATION FORM

We wish to draw the attention of the Editor to the following facts which may be considered as potential conflicts of interest and to significant financial contributions to this work.

K. M. and A. L. are employees of Proteros Biostructures GmbH. S.-L. T., H. H., W. P. and X. J. are employees of Merck, KGaA or its subsidiary, EMD Serono. P. S. is supported by a research grant from EMD Serono, a subsidiary of Merck, KGaA.

We confirm that the manuscript has been read and approved by all named authors and that there are no other persons who satisfied the criteria for authorship but are not listed. We further confirm that the order of authors listed in the manuscript has been approved by all of us.

We confirm that we have given due consideration to the protection of intellectual property associated with this work and that there are no impediments to publication, including the timing of publication, with respect to intellectual property. In so doing we confirm that we have followed the regulations of our institutions concerning intellectual property.

We understand that the Corresponding Author is the sole contact for the Editorial process (including Editorial Manager and direct communications with the office). He/she is responsible for communicating with the other authors about progress, submissions of revisions and final approval of proofs. We confirm that we have provided a current, correct email address which is accessible by the Corresponding Author and which has been configured to accept email from xuliang.jiang@emdserono.com.

Signed by all authors as follows:

Name	Signature	Date
Klaus Maskos	[illegible]	September 21, 2015
Alfred Lammens	[illegible]	September 21, 2015
Seng-Lai Tan		
Henry Hess		
Wolf Palinsky		
Pascal Schneider		
Xuliang Jiang		

AUTHOR DECLARATION FORM

We wish to draw the attention of the Editor to the following facts which may be considered as potential conflicts of interest and to significant financial contributions to this work.

K. M. and A. L. are employees of Proteros Biostructures GmbH. S.-L. T., H. H., W. P. and X. J. are employees of Merck, KGaA or its subsidiary, EMD Serono. P. S. is supported by a research grant from EMD Serono, a subsidiary of Merck, KGaA.

We confirm that the manuscript has been read and approved by all named authors and that there are no other persons who satisfied the criteria for authorship but are not listed. We further confirm that the order of authors listed in the manuscript has been approved by all of us.

We confirm that we have given due consideration to the protection of intellectual property associated with this work and that there are no impediments to publication, including the timing of publication, with respect to intellectual property. In so doing we confirm that we have followed the regulations of our institutions concerning intellectual property.

We understand that the Corresponding Author is the sole contact for the Editorial process (including Editorial Manager and direct communications with the office). He/she is responsible for communicating with the other authors about progress, submissions of revisions and final approval of proofs. We confirm that we have provided a current, correct email address which is accessible by the Corresponding Author and which has been configured to accept email from xuliang.jiang@emdserono.com.

Signed by all authors as follows:

**Name**	**Signature**	**Date**
Klaus Maskos		
Alfred Lammens		
Seng-Lai Tan		
Henry Hess		
Wolf Palinsky	WPalinski	23.09.2015
Pascal Schneider		
Xuliang Jiang		

AUTHOR DECLARATION FORM

We wish to draw the attention of the Editor to the following facts which may be considered as potential conflicts of interest and to significant financial contributions to this work.

> K. M. and A. L. are employees of Proteros Biostructures GmbH. S.-L. T., H. H., W. P. and X. J. are employees of Merck, KGaA or its subsidiary, EMD Serono. P. S. is supported by a research grant from EMD Serono, a subsidiary of Merck, KGaA.

We confirm that the manuscript has been read and approved by all named authors and that there are no other persons who satisfied the criteria for authorship but are not listed. We further confirm that the order of authors listed in the manuscript has been approved by all of us.

We confirm that we have given due consideration to the protection of intellectual property associated with this work and that there are no impediments to publication, including the timing of publication, with respect to intellectual property. In so doing we confirm that we have followed the regulations of our institutions concerning intellectual property.

We understand that the Corresponding Author is the sole contact for the Editorial process (including Editorial Manager and direct communications with the office). He/she is responsible for communicating with the other authors about progress, submissions of revisions and final approval of proofs. We confirm that we have provided a current, correct email address which is accessible by the Corresponding Author and which has been configured to accept email from xuliang.jiang@emdserono.com.

Signed by all authors as follows:

Name	Signature	Date
Klaus Maskos		
Alfred Lammens		
Seng-Lai Tan		
Henry Hess	[illegible]	Sep 23, 2015
Wolf Palinsky		
Pascal Schneider		
Xuliang Jiang		
